# CyExpDB: a web-based multi-species tissue-specific gene expression platform for functional genomics in Cyprinidae fish

**DOI:** 10.1093/database/baaf087

**Published:** 2025-12-31

**Authors:** Princy Saini, Kunal Chaudhary, Jutan Das, Naina Kumari, Sarika Jaiswal, Kiran D Rasal, U B Angadi, Mir Asif Iquebal, Dinesh Kumar

**Affiliations:** Division of Agricultural Bioinformatics, ICAR-Indian Agricultural Statistics Research Institute, Library Avenue, PUSA, New Delhi 110012, India; Graduate School, ICAR-Indian Agricultural Research Institute, Library Avenue, PUSA, New Delhi 110012, India; Division of Agricultural Bioinformatics, ICAR-Indian Agricultural Statistics Research Institute, Library Avenue, PUSA, New Delhi 110012, India; Division of Agricultural Bioinformatics, ICAR-Indian Agricultural Statistics Research Institute, Library Avenue, PUSA, New Delhi 110012, India; Division of Agricultural Bioinformatics, ICAR-Indian Agricultural Statistics Research Institute, Library Avenue, PUSA, New Delhi 110012, India; Division of Agricultural Bioinformatics, ICAR-Indian Agricultural Statistics Research Institute, Library Avenue, PUSA, New Delhi 110012, India; Fish Genetics and Biotechnology, ICAR-Central Institute of Fisheries Education, Mumbai, Maharashtra 400061, India; Division of Agricultural Bioinformatics, ICAR-Indian Agricultural Statistics Research Institute, Library Avenue, PUSA, New Delhi 110012, India; Division of Agricultural Bioinformatics, ICAR-Indian Agricultural Statistics Research Institute, Library Avenue, PUSA, New Delhi 110012, India; Division of Agricultural Bioinformatics, ICAR-Indian Agricultural Statistics Research Institute, Library Avenue, PUSA, New Delhi 110012, India

## Abstract

Freshwater fish play a vital role in global food security and sustainable development, with the Cyprinidae family standing out due to its remarkable diversity and economic importance. Despite the increasing availability of high-quality genomic resources, comprehensive transcriptomic data for major Cyprinidae species remain limited, hindering progress in functional genomics and breeding. To address this, we systematically analysed 1582 RNA-seq samples from 190 BioProjects, covering 107 tissues and cell types across five key species: *Cyprinus carpio, Carassius gibelio, Carassius auratus, Labeo rohita*, and *Ctenopharyngodon idella*. This large-scale integration of transcriptomic data led to the development of CyExpDB (https://cyexpdb.abrl.in/), a relational gene expression database built on a robust scalable relational database architecture. CyExpDB offers a user-friendly interface for retrieving, filtering, and visualizing both coding and non-coding gene expression profiles, with advanced features for exploring tissue-specific genes, functional annotations, and pathway enrichment. Users can identify tissue-specific genes using the tau score, examine their biological roles through Gene Ontology (GO) and Kyoto Encyclopedia of Genes and Genomes (KEGG) analyses, and compare expression patterns across tissues and species. CyExpDB offers an accessible resource for researchers in aquaculture, evolutionary biology, and molecular breeding. By enabling in-depth transcriptomic exploration, CyExpDB will accelerate discoveries in fish biology, support trait improvement, and inform conservation efforts.

**Database URL:**  https://cyexpdb.abrl.in/

## Introduction

Global aquatic ecosystems are increasingly threatened by a combination of climate change, habitat degradation, pollution, and overexploitation, all of which compromise biodiversity and the long-term sustainability of aquatic food systems [1, 2]. In response to these challenges, freshwater aquaculture has emerged as a vital strategy for enhancing food and nutritional security while alleviating poverty [2, 3]. The family Cyprinidae, which includes fish such as minnows and carps, is crucial for freshwater environments. It is the largest group of freshwater fish, with over 3000 different species found in places like North America, Europe, Africa, and Asia [4, 5]. Cyprinids are highly valued in both capture and culture-based fisheries, often dominating in terms of numerical abundance and biomass [6–8]. Their ecological contributions span ecosystem structuring, productivity enhancement, and functional biodiversity [9, 10]. In addition to their ecological roles, cyprinids serve as a vital and affordable source of high-quality animal protein and essential micronutrients including iron, zinc, and omega-3 fatty acids, which helps to meet the world’s nutritional demands [11, 12]. Over the past 60 years, fish consumption has dramatically increased, rising from about 9 kg per person in 1961 to over 20 kg in 2020. As a result, fish now accounts for nearly one-fifth of the animal protein consumed globally [1].

The adaptability of cyprinids to varied environmental conditions, their high feed conversion efficiency, and their compatibility with polyculture systems underscore their importance in advancing sustainable aquaculture development. Carp production in Asia has seen remarkable growth, with species such as rohu, grass carp, silver carp, common carp, catla, bighead carp, and black carp reaching a combined output of 27.6 million metric tons (MMT) in 2020. These species collectively contribute approximately 50.6% of the total inland aquaculture finfish production, highlighting their economic and nutritional value on a global scale [1, 13]. As fish play important roles in human nutrition, medicine, and ecological studies, and are often used as bio-indicators to assess the health of aquatic ecosystems, there is an increasing need to characterize their gene expression repertoires and understand the evolutionary histories of their genes [14].

Recent progress in genomics, together with the development of more affordable sequencing technologies, has greatly expanded the volume of biological data available for scientific research [15]. RNA sequencing (RNA-seq) has become a robust and widely used method for analysing gene expression throughout the entire cellular transcriptome. The transcriptome encompasses both coding and non-coding RNAs that are transcribed in cells, tissues, or organs under normal or pathological conditions. It is primarily defined by mRNA expression, reflecting the genes that are actively being expressed at any given time and offering valuable insights into gene function and regulation. By analysing transcriptome data, researchers can gain deeper insights into gene function, regulation, and cellular responses [16, 17]. Transcriptomic experiments provide dynamic insights into gene expression at the tissue level, offering valuable information on gene function and regulation. Understanding how gene expression differs across tissues is essential for elucidating the roles individual tissues play in overall physiology, metabolic activity, and trait development. Tissue-specific genes (TSGs), which display significantly higher expression in one tissue compared to others, are key to unravelling the intricacies of organismal development, tissue specialization, and evolutionary adaptations [18]. Tissue-specific expression profiles have been characterized in various aquaculture species, including Atlantic salmon (*Salmo salar*) [19], crucian carp (*C. carassius*) [20], and rainbow trout (*Oncorhynchus mykiss*) [21], providing valuable insights into species-specific transcriptional regulation. Moreover, gene expression data are crucial for understanding the genetic adaptability of aquaculture species, enabling the identification of genes linked to important traits such as heat tolerance, disease resistance, and feed efficiency [22]. Understanding these molecular mechanisms is fundamental for developing precision breeding strategies aimed at enhancing resilience and productivity in aquaculture systems.

Furthermore, transcriptome-based databases and web servers, developed through advances in RNA-seq technologies, have become indispensable resources for exploring gene expression dynamics and the molecular mechanisms underlying a wide range of biological processes. To improve accessibility and utility, comprehensive platforms have been established that integrate sequencing data with functional annotations and genome mapping information. Resources such as Expression Atlas [23], GXD [24], and GeneFriends [25] provide valuable gene expression data, primarily focusing on humans and established model organisms. To address the need for fish-specific resources, specialized platforms like FishGET [26] and PhyloFish [27] have been developed. FishGET compiled 1362 paired-end RNA-seq samples from 97 BioProjects across eight teleost species, including data for *Ctenopharyngodon idella* from 43 BioProjects. In contrast, our study analysed a much larger dataset for *C. idella*, integrating RNA-seq data from 88 BioProjects, thereby providing a more comprehensive and TSG expression resource for this species. PhyloFish, on the other hand, provides de novo transcriptomes and gene expression profiles across 10 tissues from 23 ray-finned fish species, but does not include several major cyprinid species, namely *Cyprinus carpio, Labeo rohita, Carassius gibelio, Carassius auratus*, or *Ctenopharyngodon idella*. While both platforms are valuable, they lack integration with large-scale RNA-seq datasets and do not support TSG analysis or cross-species comparisons within the Cyprinidae family. Moreover, the absence of tissue-specificity metric calculations limits the scope of tissue-level functional genomics research. However, no dedicated expression atlas currently exists that integrates high-resolution, TSG expression data across multiple Cyprinidae species. This gap highlights the need for a user-friendly, comprehensive web-based resource to support functional genomics and breeding in this important fish family. A centralized, high-resolution expression resource for Cyprinidae will not only accelerate functional genomics research but also support selective breeding programmes aimed at improving aquaculture productivity and sustainability. Such a database can facilitate the discovery of candidate genes associated with economically important traits, stress adaptation, and disease resistance.

To address this gap, we developed CyExpDB (https://cyexpdb.abrl.in/), a comprehensive and user-friendly gene expression atlas tailored for five major Cyprinidae species: *C. carpio, C. gibelio, C. auratus, L. rohita*, and *C. idella*. The database incorporates 1582 RNA-seq datasets from 190 BioProjects, encompassing 107, with data mapped to their respective high-quality genome assemblies (*C. carpio*: GCF_018340385.1, *L. rohita*: GCF_022985175.1, *C. gibelio*: GCF_023724105.1, *C. idella*: GCF_019924925.1, and *C. auratus*: GCF_003368295.1). CyExpDB provides TSG expression patterns and developmental dynamics across these species, enabling researchers to investigate gene regulatory networks, evolutionary adaptations, and functional traits critical for aquaculture and ecological studies.

## Materials and methods

### Data acquisition and preprocessing

**Figure 1.**
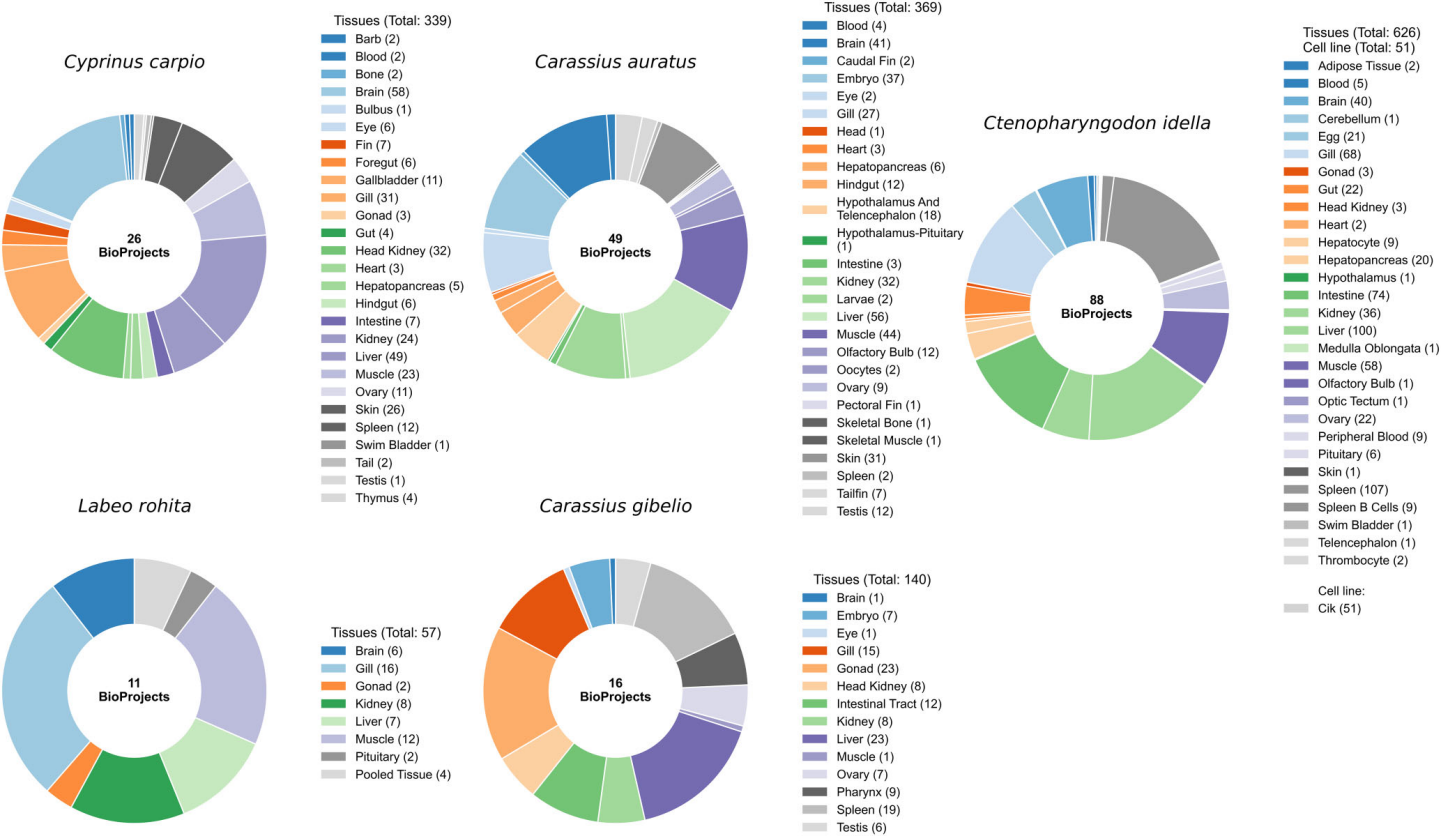
Tissue diversity and sample counts in cyprinid species. Donut charts illustrate the number of BioProjects and the distribution of RNA-seq samples across different tissues for each of the five major Cyprinidae species included in this study. The charts highlight the extensive tissue diversity and sampling depth achieved, providing a robust foundation for comprehensive gene expression profiling.

RNA-seq datasets were retrieved from the NCBI Sequence Read Archive (SRA) [28] using the SRA Toolkit for five major Cyprinidae species: *C. carpio, L. rohita, C. gibelio, C. idella*, and *C. auratus*. Corresponding reference genome assemblies (FASTA) and gene annotation files (GTF/GFF) were downloaded from NCBI. A total of 190 BioProjects were included in this study, distributed as follows: 26 for *C. carpio*, 11 for *L. rohita*, 16 for *C. gibelio*, 88 for *C. idella*, and 49 for *C. auratus*. Altogether, these BioProjects encompass 1582 RNA-seq samples representing 107 distinct biological sources, providing a comprehensive resource for gene expression profiling across the Cyprinidae family. To ensure consistency and reproducibility, all samples were curated and classified as either tissues or cell lines using standardized vocabularies (BRENDA Tissue Ontology) [29]. These categories are incorporated into CyExpDB to enable tissue and cell line-specific filtering and analysis. A summary of genome assemblies, BioProjects, RNA-seq samples, and tissue diversity for each species is provided in Table 1. Detailed metadata for all RNA-seq samples, including tissue source, assay type, BioProject, BioSample, experimental conditions, and developmental stage, are presented in Supplementary Table S1. Figure 1 illustrates the distribution of BioProjects and the number of RNA-seq samples across tissues and cell lines for the five Cyprinidae species, highlighting the extensive biological and experimental diversity represented in CyExpDB.

**Table 1.** Overview of genome assemblies, BioProjects, RNA-seq samples, and tissue types analysed across five Cyprinid species.

Species	Assembly	Number of BioProjects	Number of samples	Number of tissues
*Cyprinus carpio*	GCF_018340385.1	26	339	28
*Labeo rohita*	GCF_022985175.1	11	57	8
*Carassius gibelio*	GCF_023724105.1	16	140	14
*Ctenopharyngodon idella*	GCF_019924925.1	88	677	30
*Carassius auratus*	GCF_003368295.1	49	369	27
Total		190	1582	107

After downloading, raw reads underwent a standardized preprocessing workflow to ensure data quality and consistency. Quality assessment was performed using FastQC [30], and adapter sequences as well as low-quality bases were trimmed using Trimmomatic (v0.39) [31]. Reads shorter than 40 bp, with an average Phred score below 30, or showing excessive sequencing noise were discarded. To evaluate the overall effectiveness of the preprocessing pipeline, a comprehensive QC report was generated that included read depth, mapping rate distributions. This rigorous workflow ensured the retention of only high-quality, biologically reliable data suitable for accurate downstream expression quantification.

### Mapping and quantification

High-quality reads were mapped to their respective reference genomes. Reference genome indices were constructed using HISAT2 (v2.2.1) [32] with the hisat2-build utility. Clean reads from each sample were aligned to the corresponding reference genome using HISAT2 with the ‘--dta’ option. The resulting SAM files were converted to compressed BAM format using Samtools (v1.9) [33], followed by sorting and indexing to facilitate efficient downstream analysis.

**Figure 2.**
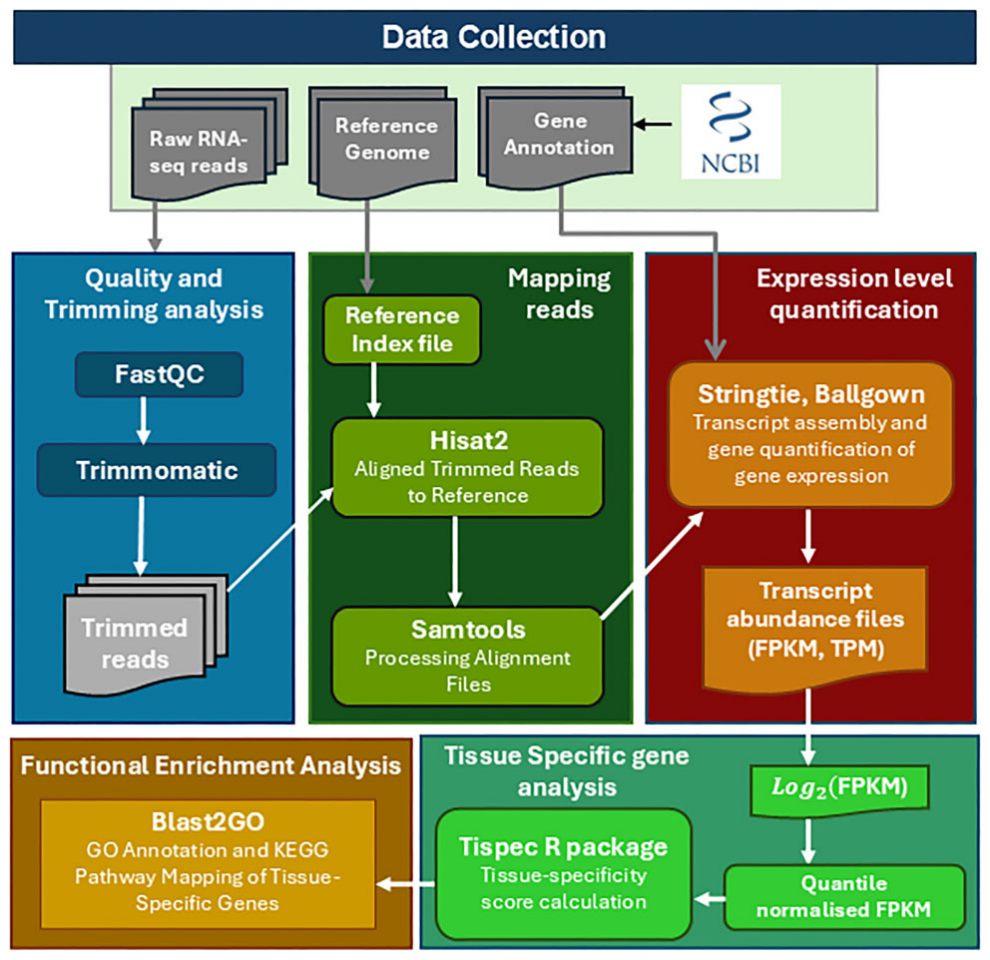
Workflow for the construction of the Cyprinidae Expression Atlas. A schematic overview of the analytical pipeline, detailing the key steps from raw RNA-seq data preprocessing, alignment to reference genomes, expression quantification (FPKM/TPM), coding potential estimation, functional annotation, and tissue-specificity analysis, culminating in the development of a comprehensive gene expression atlas for Cyprinidae species.

StringTie (v2.1.4) [34] was employed for transcript assembly and expression quantification in each dataset. It simultaneously reconstructs transcript structures and quantifies gene expression levels, reported as Fragments Per Kilobase of transcript per Million mapped reads (FPKM) and Transcripts Per Million (TPM). To ensure quantification was restricted to reference-annotated transcripts, StringTie was executed with the -e and -G options, while the -A option was used only to generate a gene-level abundance summary. The coding potential of annotated transcripts was evaluated using CPC2, which distinguishes coding from non-coding genes based on sequence-derived features such as open reading frame length, Fickett score, and isoelectric point [35]. The complete workflow for the construction of the Cyprinidae Expression Atlas, from raw RNA-seq read processing through alignment, expression quantification, coding potential estimation, functional annotation, and tissue-specificity analysis, is illustrated in Fig. 2.

### Tissue-specificity index for construction of gene expression atlas

Identification of TSGs across 107 tissues was performed using the tau (τ) index, implemented via the ‘tispec’ v0.99 package in R (v4.1.2) [36, 37]. The τ index, which ranges from 0 to 1, provides a robust measure of tissue specificity and is widely recognized for its effectiveness in detecting evolutionarily conserved TSGs. For this analysis, FPKM values were used as the expression metric, as they account for both sequencing depth and transcript length, enabling unbiased comparisons across samples. After adding a pseudocount of 1, the FPKM values were log₂-transformed to stabilize variance, followed by batch correction using the ComBat algorithm from the sva R package [38], which applies an empirical Bayes framework to minimize technical variation while preserving biological differences [39]. Principal Component Analysis (PCA) of the ComBat-adjusted matrix revealed distinct tissue-specific clustering without batch-associated grouping, confirming effective correction (Supplementary Fig. S1).

Following batch correction, replicate samples were averaged within each tissue to generate tissue-level expression matrices, which were subsequently subjected to quantile normalization prior to τ computation [40]. FPKM-based normalization remains a robust and widely adopted approach in large-scale transcriptomic analyses. Numerous studies have successfully computed τ or related tissue-specificity indices using log-transformed FPKM values, including the Strawberry transcriptome atlas [41], human expression datasets [42] *Mus musculus* [43], and *Brassica rapa* [44]. These studies collectively demonstrate that when normalization is applied consistently, particularly through log transformation and quantile scaling, FPKM values provide reliable and comparable measures of gene expression suitable for cross-tissue analyses. Based on the τ index, genes were categorized into three groups: absolute/highly specific genes (HSGs) (τ ≥ 0.85), intermediate-specific genes (0.2 ≤ τ < 0.8), and housekeeping/low-specific genes (τ < 0.2). To assess the robustness of tissue-specificity estimates, τ values were computed across different normalization strategies (FPKM and TPM) for all five Cyprinidae species. The results demonstrated high concordance, confirming that tissue-specificity patterns were consistent regardless of normalization approach (Supplementary Fig. S2). The τ index for each gene was calculated as:


\begin{eqnarray*}
\tau = \,\,\frac{{{\mathrm{\Sigma }}_{i = 1\,\,}^{\mathrm{N}}\left( {1\,\, - \,\,{x_i}} \right)}}{{{\mathrm{N}} - 1}}
\end{eqnarray*}

where N is the total number of tissues analysed, and ${x_i}$ is the normalized expression specificity score of the gene in tissue *i*. This calculation yields a tau index value between 0 (broad expression across tissues) and 1 (expression restricted to one or a few tissues) [45, 46]. This classification enables a nuanced understanding of tissue-specific expression patterns and supports the identification of genes with critical roles in specific tissues, thereby providing valuable insights into functional genomics and biomarker discovery [40]. Genes with a τ ≥ 0.85 are considered to exhibit specific expression, indicating predominant expression in one or a few tissues, whereas genes with a tau score < 0.2 are categorized as having widespread expression across multiple tissues.

### Functional annotation and enrichment analysis

Functional annotation of the identified genes was performed using Blast2GO [47], with default parameters, focusing on Gene Ontology (GO) and Kyoto Encyclopedia of Genes and Genomes (KEGG) pathway analyses. GO annotation was performed to assign genes to the three main categories: biological processes, molecular functions, and cellular components. Sequence similarity searches were carried out using the standalone BLASTX tool against the NCBI non-redundant (nr) protein database to support gene function assignments. Subsequently, KEGG pathway mapping was carried out to identify associated metabolic and signalling pathways [48]. To enable cross-species comparison, orthologous genes among the five Cyprinidae species were identified using OrthoFinder (v2.5.4) [49] with DIAMOND for all-vs-all sequence similarity searches. The resulting orthogroups were incorporated into the Ortholog Gene Browser for comparative analysis across species. Ortholog mapping and tissue ontology tables used for cross-species harmonization are available for download in the Download section of CyExpDB.

### Development of Cyprinidae Expression Atlas

**Figure 3.**
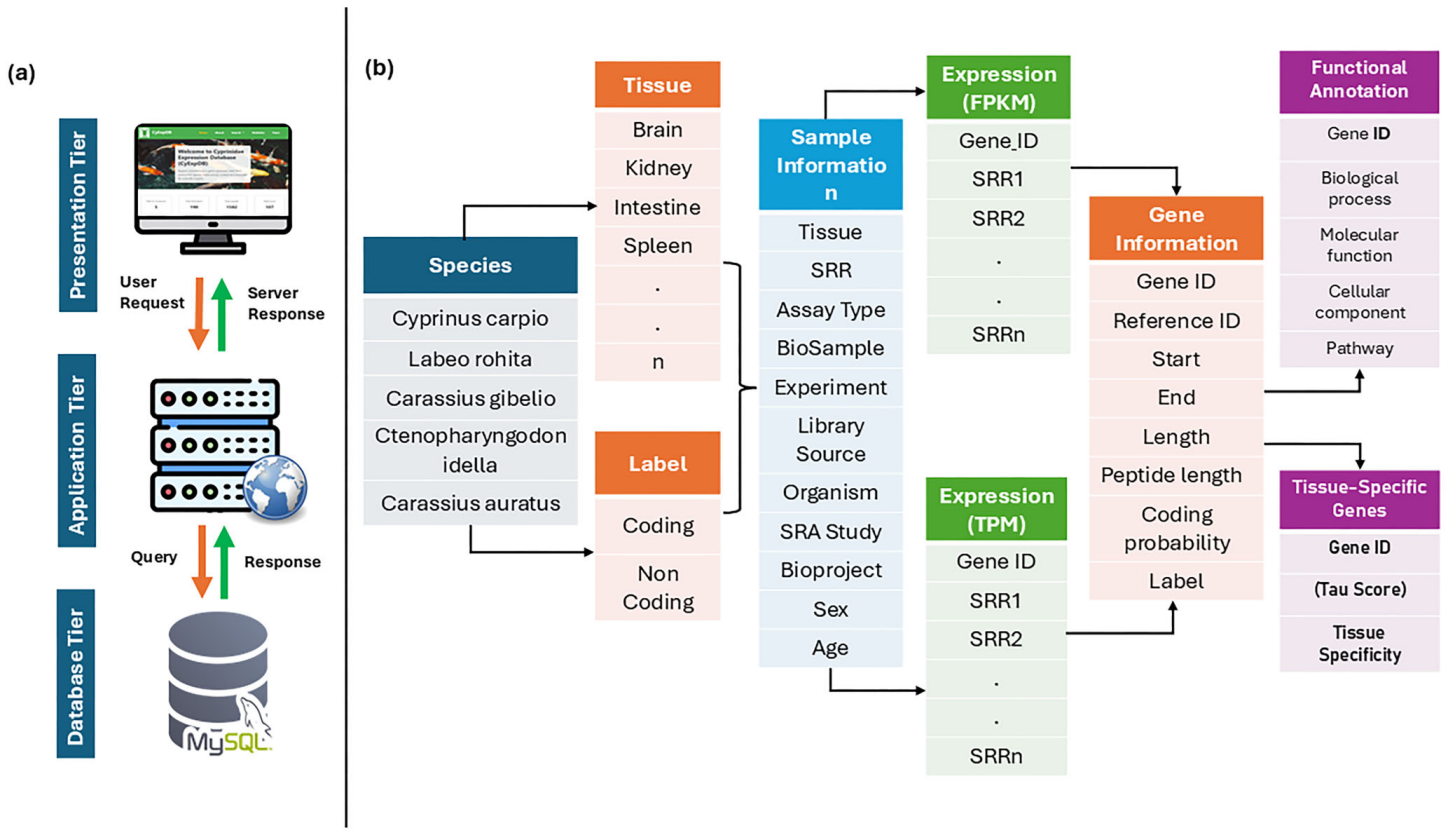
Three-tier architecture and data structure of CyExpDB. A schematic representation of the CyExpDB relational database, showing its three-tier architecture (presentation, application, and data layers) and the integration of tissue-specific gene expression data across Cyprinidae species.

To facilitate access to comprehensive gene expression profiles across multiple tissues and Cyprinidae species, we developed CyExpDB, a user-friendly, tissue-wise expression atlas. CyExpDB is implemented as a relational database using a three-tier architecture, which consists of presentation (client), application (logic), and data (database) layers. The presentation layer was built using HTML, CSS, Bootstrap 5.0, JavaScript, and PHP (https://www.php.net/), providing an interactive and intuitive web interface for users. The application layer, implemented using PHP, manages the server-side logic and interactions with the database, while the database layer utilizes a MySQL server (https://www.mysql.com/) for robust and scalable data management. CyExpDB integrates gene expression data from 1582 RNA-seq datasets, encompassing 107 tissues across five major Cyprinidae species. Figure 3 illustrates the three-tier architecture and data structure of CyExpDB, highlighting its design as a relational database for TSG expression in Cyprinidae.

## Results and discussion

### Preprocessing, alignment, and expression profiling

The Cyprinidae Expression Database (CyExpDB) was developed by integrating 1582 RNA-seq samples from 190 BioProjects, representing five major Cyprinidae species: *C. carpio, C. gibelio, C. auratus, L. rohita*, and *C. idella*. Stringent quality control was performed on all raw RNA-seq reads, including the removal of adapter sequences and low-quality bases removal, ensuring only high-quality data were retained for downstream analysis. High-quality reads were aligned to their respective reference genomes using optimized parameters to maximize mapping accuracy and minimize misalignments. Across all samples, a total of 305 459 genes were identified, comprising 90 497 coding genes and 214 962 non-coding genes. Gene expression levels were normalized using both FPKM and TPM methods, enabling accurate comparisons across different tissues and species. Analysis revealed that among the five Cyprinidae species, the proportion of coding genes ranged from 18.4% in *C. gibelio* to 45.8% in *C. idella*. Specifically, *L. rohita* had 29.9% coding and 70.1% non-coding genes, *C. gibelio* had 18.4% coding and 81.6% non-coding genes, *C. auratus* had 36.5% coding and 63.5% non-coding genes, *C. idella* had 45.8% coding and 54.2% non-coding genes, and *C. carpio* had 29.8% coding and 70.2% non-coding genes. This predominance of non-coding genes highlights the complexity and regulatory potential of Cyprinidae transcriptomes. Table 2 presents the distribution of coding and non-coding genes across the five Cyprinidae species. It was observed that, among these species, the proportion of coding genes ranged from 18.4% in *C. gibelio* to 45.8% in *C. idella*, while non-coding genes accounted for 54.2%–81.6%. The predominance of non-coding genes in all species aligns with trends observed in other organisms and highlights the regulatory complexity and functional significance of non-coding elements within Cyprinidae genomes.

**Table 2.** Summary of genome assembly details and gene content for five Cyprinidae species.

Species	Assembly accession	Assembly size (mb)	Assembly level	Coding genes	Non-coding genes
*Cyprinus carpio*	GCF_018340385.1	1700	Chromosome	17 751	41 828
*Labeo rohita*	GCF_022985175.1	1100	Chromosome	9463	22 237
*Carassius gibelio*	GCF_023724105.1	1600	Chromosome	18 191	80 538
*Ctenopharyngodon idella*	GCF_019924925.1	893.2	Chromosome	14 565	17 212
*Carassius auratus*	GCF_003368295.1	1800	Chromosome	30 527	53 147

### Tissue-specific gene identification and classification

Tissue-specific gene identification is essential for characterizing the molecular basis of specialized tissue functions and for facilitating the discovery of tissue-specific biomarkers [17]. In this study, we classified genes from each of the five Cyprinidae species into three categories—highly specific, intermediate, and housekeeping—based on their tau (τ) scores, a quantitative measure of tissue specificity. The τ score was calculated for each gene to quantify its specificity, enabling a detailed examination of gene expression patterns across different tissues and samples.

**Figure 4.**
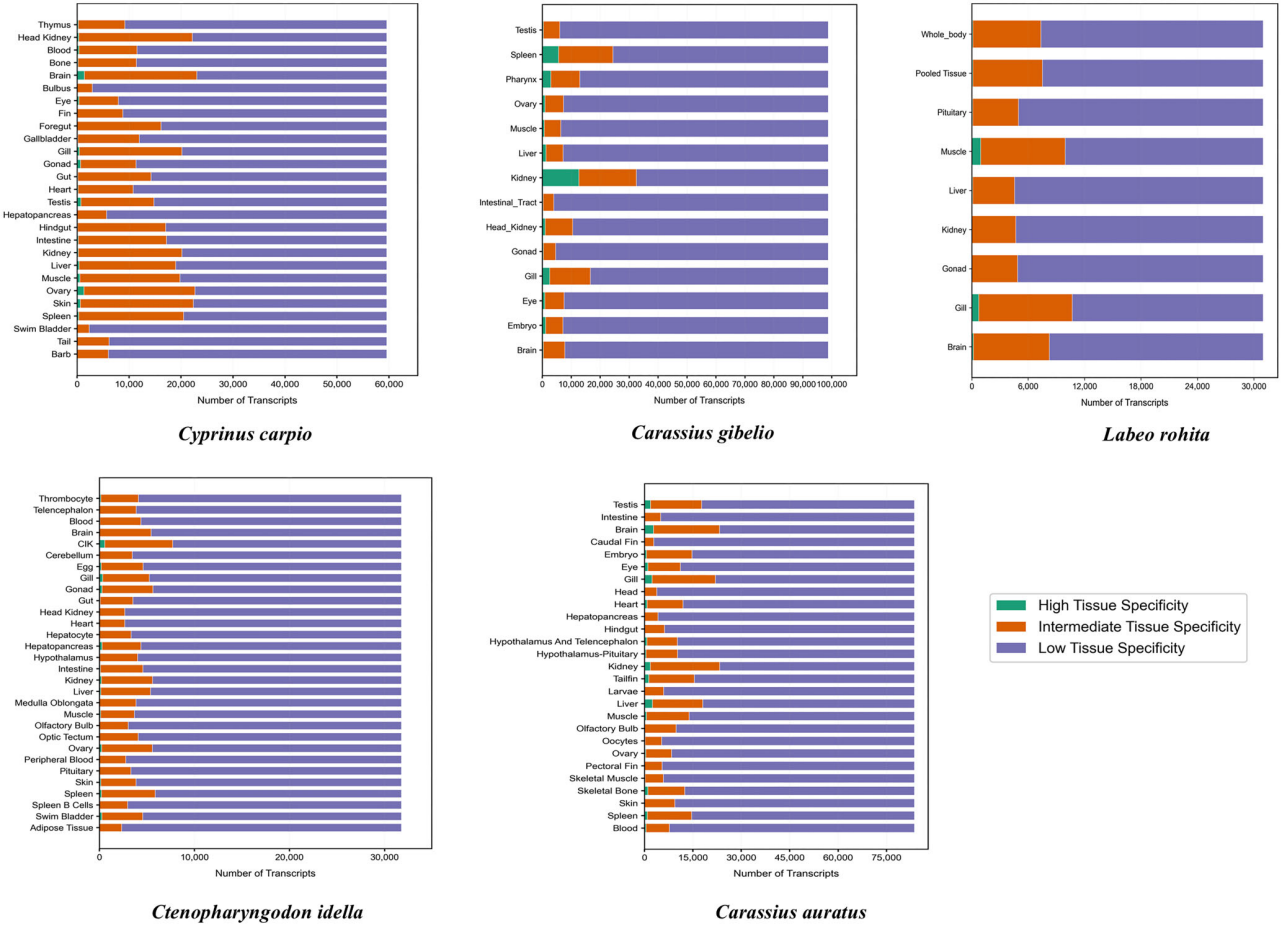
Histogram showing the distribution of highly specific, intermediate, and housekeeping genes across the five major Cyprinidae species, based on tau (τ) score classification.

The distribution of gene categories across species is graphically illustrated in Fig. 4, which presents a histogram of highly specific, intermediate, and housekeeping genes for each fish species. Across all tissues analysed, the number of genes classified as highly specific (τ > 0.8) and intermediate specific (0.2 ≤ τ < 0.8) varied considerably among the five Cyprinidae species. In *L. rohita*, 2418 genes were identified as highly specific and 60 454 as intermediate specific. *Cyprinus carpio* exhibited a greater number of both highly specific (9259) and intermediate-specific genes (366 166). Similarly, *C. gibelio* displayed 25 835 highly specific and 118 521 intermediate-specific genes. In *C. idella*, 3940 genes were highly specific and 122 745 were intermediate specific, while *C. auratus* showed 21 430 highly specific and 278 117 intermediate-specific genes across all tissues examined.

Analysis of TSG expression revealed notable differences among tissues within each species. For example, in *C. auratus*, the brain exhibited the highest number of HSGs (2849), followed by the liver (2486) and gill (2378), while the caudal fin and olfactory bulb displayed the lowest numbers (85 and 65, respectively). In *C. idella*, the CIK (*Ctenopharyngodon idella* Kidney) cell line had the highest count of HSGs (567), followed by the spleen (231), swim bladder (269), and ovary (248), whereas the optic tectum and telencephalon had the lowest (6 and 10 genes, respectively). For *C. gibelio*, the head kidney exhibited the greatest number of HSGs (4 498), followed by muscle (3783) and pharynx (2976), while the intestinal tract (304) and brain (371) showed the lowest numbers. In *C. carpio*, the brain contained the highest number of HSGs (1389), closely followed by the ovary (1336) and testis (761), whereas the tail (25), swim bladder (31), and bulbus (29) had the fewest. In *L. rohita*, muscle exhibited the highest number of HSGs (960), followed by gill (742) and brain (178), while the gonad (43), kidney (50), and pituitary (94) displayed the lowest counts.

Functional enrichment analysis of highly specific TSGs revealed that tissues within the same functional group often shared similar sets of enriched GO terms, reflecting conserved biological roles. For instance, in *L. rohita*, brain-specific genes were predominantly enriched in GO terms related to neurodevelopment and transcriptional regulation, such as brain development (GO:0007420), neuron differentiation (GO:0030182), regulation of transcription by RNA polymerase II (GO:0006357), cell fate specification (GO:0001708), midbrain development (GO:0030901), midbrain–hindbrain boundary development (GO:0030917), axon development (GO:0061564), forebrain neuron development (GO:0021884), chemokine-mediated signalling pathway (GO:0070098), neuropeptide signalling pathway (GO:0007218), and cell proliferation in the hindbrain (GO:0021534), mediated by key neural regulators like fezf1, gbx2, en2b, zic1, bhlhe22, pou4f2, irx5b, lbx1b, otpb, zbtb18, and neurod6b (τ ≥ 0.8) [50–56]. In contrast, kidney-specific TSGs were predominantly associated with mitochondrial energy metabolism and ion transport, including electron transport chain (GO:0022900), oxidative phosphorylation (GO:0006119), mitochondrial electron transport (GO:0006120), ATP synthesis-coupled electron transport (GO:0042773), and proton transmembrane transport (GO:1902600), driven by high expression of respiratory chain components and ion transporters such as slc34a1b [57]. In *C. carpio*, brain-specific TSGs were enriched for G protein-coupled receptor (GPCR) signalling pathway (GO:0007186), sensory perception of smell (GO:0007608), signal transduction (GO:0007165), and neuropeptide signalling (GO:0007218), with key genes such as apc2, arhgap39, lbx1a, asic1a, astn1, ajap1, and asphd1 showing strong tissue specificity [58–63]. Similarly, in *C. auratus*, enrichment was observed for sensory perception of smell (GO:0007608), signal transduction (GO:0007165), and stabilization of membrane potential (GO:0030322), with highly brain-specific genes including [64, 65]. In *C. idella*, kidney tissues showed enrichment for GPCR signalling (GO:0007186) and transmembrane transport (GO:0055085), with the highly TSG slc22a7b.2 encoding an organic ion transporter, and in liver tissue, foxl3 was associated with regulation of transcription by RNA polymerase II (GO:0006357) and phosphorylation (GO:0016310) [66]. Ortholog analysis revealed conserved brain-specific expression of the *lbx1a* orthogroup (OG0018250) across Cyprinidae species. *Cyprinus carpio* and its orthologs in *C. auratus* (τ = 0.814) and *L. rohita* (τ = 0.875) showed strong brain enrichment, whereas *C. gibelio* (τ = 0.501) and *C. idella* (τ = 0.259) exhibited moderate specificity, reflecting partial conservation of neural function.

### Development of Cyprinidae Expression Database

CyExpDB, the Cyprinidae Expression Database, is a pioneering and comprehensive gene expression resource developed using a robust three-tier architecture and dedicated to the study of five major Cyprinidae species: *C. carpio, C. gibelio, C. auratus, L. rohita*, and *C. idella*. The database integrates 1582 RNA-seq samples from 190 BioProjects, covering 107 distinct tissues and cell types, and provides users with the ability to retrieve both coding and non-coding gene expression profiles across tissues. CyExpDB features several interactive sections, including Home, About, Search, Statistics, Team, Download, and Help. The Home page offers a concise overview of the database, complete with a Quick Start guide and direct links to species-specific datasets. The About page describes the data sources and details the quality control and analytical methods used for processing and annotating the RNA-seq data. In the Search section, users can access a dropdown menu with hyperlinks that enable detailed exploration of gene and sample information, expression values in FPKM and TPM, and functional annotations, all organized in intuitive tables with advanced filtering and search capabilities. The Statistics section presents graphical summaries of the dataset, including the distribution of BioProjects, samples, and tissues for each species, facilitating a comprehensive understanding of the data landscape. The Team page highlights the individuals involved in the development and curation of CyExpDB, along with their contact information. The Download section provides access to normalized TPM matrices, tissue-specificity (τ) scores, cross-species ortholog mapping tables, tissue ontology vocabularies, and a comprehensive quality-control summary. The *Help* section provides documentation and step-by-step guidance for data interpretation and use in downstream analyses.

Each species page in CyExpDB provides a summary that includes the scientific name, genome assembly with a direct link to NCBI, chromosome number, genome size, and other relevant genomic details. Users can view species images and access FTP links to download genome and annotation files in fna, gtf, and gff formats. A selection panel allows users to choose tissue type and gene class (coding or non-coding) before proceeding. After selection and submission, users are directed to a results overview page, where they can navigate to Gene Information, Sample Details, Expression (FPKM), Expression (TPM), Tissue Specific Genes, and Functional Annotation each providing targeted data and analysis options.

The Gene Information page presents key details for each gene in a clear, organized table to support efficient exploration and analysis. Each row displays the gene ID (hyperlinked to NCBI), reference sequence, start and end positions, transcript and peptide lengths, coding probability, and coding label. Functional annotations are included, such as GO terms for molecular function, biological process, and cellular component, with direct links to external GO resources where available. Pathway information is also provided and hyperlinked when applicable. Users can search for specific genes, adjust the number of rows displayed per page, and download the data as a CSV file for further analysis. This intuitive layout makes it easy to browse, filter, and interpret gene data for any selected species.

On the Sample Information page of CyExpDB, users are provided with comprehensive metadata for each RNA-seq sample, meticulously organized to facilitate efficient data exploration. Essential identifiers such as BioSample and BioProject accession numbers are prominently displayed and hyperlinked, enabling direct access to the corresponding entries on the NCBI platform for seamless cross-referencing. Each sample entry includes a detailed description that encompasses the specific tissue or cell type examined, as well as relevant contextual information such as cultivar or strain, developmental stage, treatment details (if applicable), and any other pertinent experimental attributes. This information is presented in a structured tabular format, designed for clarity and ease of navigation. The table incorporates both pagination and scrollbars—vertically and horizontally—ensuring that users can effortlessly browse and interpret large datasets. This thoughtful layout supports both targeted queries and broad overviews, making it straightforward for researchers to retrieve and analyse the precise sample information needed for their studies.

The FPKM and TPM Expression pages in CyExpDB provide an interactive platform for exploring gene expression profiles across samples. Both pages feature a searchable and filterable table, where users can enter a gene ID to quickly locate specific genes and adjust the number of rows displayed per page for easier navigation. Expression values for each gene are organized in a clear tabular format, with columns representing individual samples and rows representing gene IDs. A ‘Show Heatmap’ button allows users to visualize the selected gene expression data as an interactive heatmap, making it easy to interpret expression patterns across multiple samples. This unified interface supports both FPKM and TPM normalization methods, enabling efficient comparison, filtering, and analysis of gene expression data for comprehensive transcriptomic studies.

The Tissue Specific Genes page in CyExpDB provides detailed insights into the tissue specificity of genes based on the tau score, a measure of expression breadth across tissues. Genes are classified into three categories: highly/absolute-specific (tau score 0.8–1.0), intermediate-specific (0.2–0.8), and low-specific/housekeeping (0–0.2), with clear definitions provided at the top of the page. Users can filter genes by specificity class using dedicated buttons and search for specific gene IDs within the selected tissue. The results are displayed in a paginated table, showing the gene ID, tau score, and tissue-specificity classification, with each gene ID hyperlinked for access to further details. This interface enables efficient exploration and interpretation of tissue-enriched, intermediate, and broadly expressed genes within any selected tissue or species.

**Figure 5.**
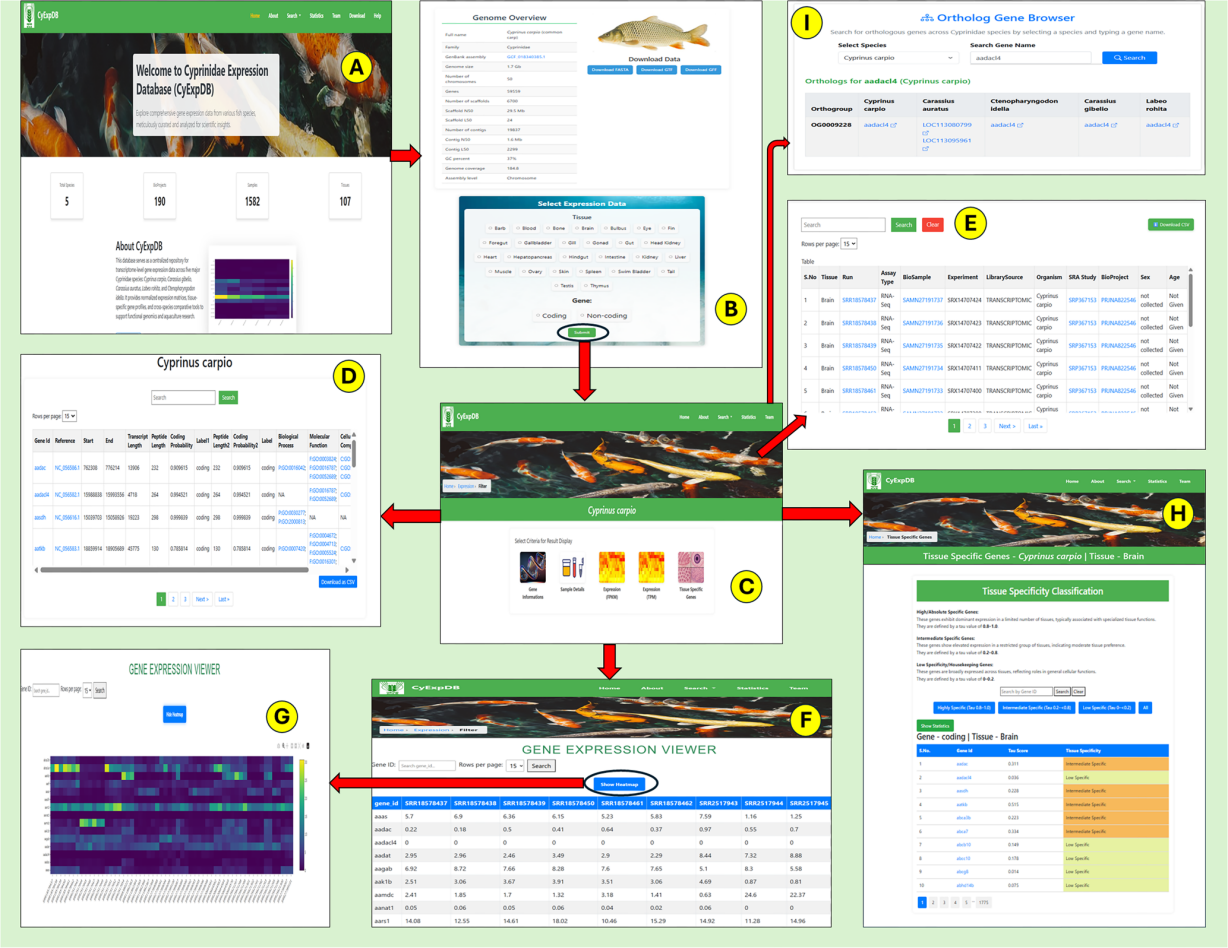
Overview and workflow of the Cyprinidae Expression Database (CyExpDB). (A) Home page providing access to database sections and species selection. (B) Genome overview page displaying species-specific genomic information and data download options, with a selection panel for tissue type and gene class. (C) Results overview page offering navigation to gene information, sample details, expression profiles, and tissue-specific gene analysis. (D) Gene Information page displaying detailed gene annotations and functional data. (E) Sample Information page summarizing metadata for each RNA-seq sample. (F) Expression Profiles page presenting gene expression values (FPKM/TPM) in a tabular format with search and filtering options. (G) Heatmap visualization of gene expression patterns across samples. (H) Tissue Specific Genes page showing classification of genes based on tau score and tissue specificity. (I) Ortholog Gene Browser enabling users to identify orthologous genes across Cyprinidae species.

As shown in Table 2, the gene content across species is substantial, with *L. rohita* containing 9463 coding and 22 237 non-coding genes, *C. gibelio* possessing 18 191 coding and 80 538 non-coding genes, *C. auratus* featuring 30 527 coding and 53 147 non-coding genes, *C. idella* comprising 14 565 coding and 17 212 non-coding genes, and *C. carpio* including 17 751 coding and 41 828 non-coding genes. This comprehensive integration of gene expression and annotation data across multiple species and tissues makes CyExpDB a valuable platform for exploring the molecular basis of tissue specialization, gene regulation, and evolutionary adaptation in Cyprinidae fishes. The overall structure, navigation workflow, and key features of CyExpDB are illustrated in Fig. 5.

### Utility of CyExpDB

CyExpDB is a comprehensive and user-friendly resource that provides detailed transcriptomic profiles for five major Cyprinidae species: *C. carpio, C. gibelio, C. auratus, L. rohita*, and *C. idella*. The database integrates gene expression information from 1582 RNA-seq samples, covering 107 tissues and cell types (Fig. 1). For each species, the database includes both coding and non-coding gene expression data (Table 2), enabling users to explore the regulatory complexity of cyprinid genomes.

Gene expression data are available in both FPKM and TPM formats, allowing for flexible and accurate cross-tissue and cross-species comparisons. Users can visualize expression profiles using interactive heatmaps or tissue-wise charts, facilitating the identification of genes with specific expression patterns or those differentially expressed across tissues. The platform also supports gene-wise visualization, allowing users to quickly determine in which tissues a gene is highly or lowly expressed.

CyExpDB facilitates the identification and exploration of TSGs, which are critical for understanding the molecular mechanisms underlying tissue specialization and for discovering potential biomarkers relevant to physiology, aquaculture, and disease resistance. The database provides downloadable lists of TSGs for each tissue, supporting downstream analyses such as functional annotation, biomarker discovery, and evolutionary studies. In addition to expression data, CyExpDB integrates functional annotations from major databases, including GO and KEGG pathways. This allows users to explore the biological significance of their findings and to link expression patterns with functional pathways and processes. To support cross-species comparative and evolutionary analyses, CyExpDB includes orthologous gene information that allows examination of conserved and divergent expression patterns across species.

The intuitive web interface of CyExpDB enables efficient browsing, searching, visualization, and downloading of gene expression datasets, annotation files, TSG lists, and ortholog resources. Users can download expression matrices and annotation files for offline analysis, making CyExpDB a valuable tool for both experimental and computational researchers in genomics, evolutionary biology, and aquaculture.

## Conclusion

This study presents a comprehensive catalogue of TSG expression in Cyprinidae, integrating globally distributed RNA-seq data into a unified and accessible platform. As the first web-based expression atlas dedicated to five major cyprinid species, CyExpDB encompasses 1582 RNA-seq samples from 107 distinct tissues and cell types (Fig. 1). The database provides both coding and non-coding gene expression profiles, enabling researchers to explore the regulatory complexity and transcriptomic diversity that underpin physiological and developmental processes in this important fish family.

CyExpDB offers an intuitive interface for retrieving and visualizing TSG expression, supporting the identification of TSGs and their associated biological pathways through integrated functional annotation. This resource empowers researchers to investigate molecular mechanisms underlying tissue specialization, environmental adaptation, and disease susceptibility in Cyprinidae. CyExpDB also includes orthologous gene information to support comparative analyses of conserved and divergent expression patterns across species. The availability of detailed expression data across a wide range of tissues and developmental stages also supports the discovery of biomarkers relevant to aquaculture, environmental adaptation, and evolutionary studies.

By delivering a centralized, high-quality transcriptomic resource, CyExpDB supports comparative analyses across species, organs, and experimental conditions, advancing research in fish genomics, selective breeding, and conservation biology. Researchers can leverage this database to deepen their understanding of gene regulation, tissue function, and trait association in cyprinids, ultimately contributing to improved aquaculture practices and the sustainable management of these ecologically and economically significant fish species.

## Supplementary Material

baaf087_Supplemental_Files

## Data Availability

All analysis scripts and workflows are available at *princysaini/CyExpDB_pipeline*. CyExpDB also provides bulk downloads of expression matrices, τ values, metadata, and ortholog tables through its Downloads section.

## References

[1] FAO . The State of World Fisheries and Aquaculture 2022: Towards blue transformation. Rome: FAO, 2022.

[2] Heras J . Fish transcriptomics: Applied to our understanding of aquaculture. In L. Z. Rodriguez-Anaya & C. M. Escobedo-Bonilla (Eds.), Transcriptomics from aquatic organisms to humans (pp. 71–90). CRC Press.. 2021;

[3] Searchinger T, Waite R, Hanson C et al. Creating a Sustainable Food Future: A menu of solutions to feed nearly 10 billion people by 2050.. World Resources Institute. Washington, DC 2019.

[4] Li X, Guo B. Substantially adaptive potential in polyploid cyprinid fishes: Evidence from biogeographic, phylogenetic and genomic studies.. Proceedings of the Royal Society B: Biological Sciences. 2020;287 (1920):20193008. 10.1098/rspb.2019.3008 PMC7031659 32075533

[5] Wang Y, Zhang X, Wang J et al. Genomic insights into the seawater adaptation in Cyprinidae. BMC Biology. 2024;22(1):87. 10.1186/s12915-024-01885-2 38637780 PMC11027309

[6] He S, Mayden RL, Wang X et al. Molecular phylogenetics of the family Cyprinidae (Actinopterygii: Cypriniformes) as evidenced by sequence variation in the first intron of S7 ribosomal protein-coding gene: Further evidence from a nuclear gene of the systematic chaos in the family. Molecular Phylogenetics and Evolution. 2008;46(3):818–29. 10.1016/j.ympev.2007.06.001 18203625

[7] Tiogué CT, Tomedi MTE, Tchoumboué J. Reproductive strategy of Labeobarbus batesii (Boulenger, 1903) (Teleostei: Cyprinidae) in the Mbô floodplain rivers of Cameroon. International Journal of Zoology. Int J Zool. 2013;Article ID 452329.

[8] Petit J, David L, Dirks R et al. Genomic and transcriptomic approaches to study immunology in cyprinids: What is next?. Dev Comp Immunol. 2017;75:48–62. 10.1016/j.dci.2017.02.022 28257855

[9] Nelson JS, Grande TC, Wilson MVH. Fishes of the World. Wiley, Hoboken, New Jersey, U.S.A., 2016. 10.1002/9781119174844

[10] Krabbenhoft TJ, Turner TF. Comparative transcriptomics of cyprinid minnows and carp in a common wild setting: a resource for ecological genomics in freshwater communities. DNA Res. 2018;25:11–23. 10.1093/dnares/dsx034 28985264 PMC5824830

[11] Jaya-Ram A, Fuad F, Zakeyuddin MS et al. Muscle fatty acid content in selected freshwater fish from Bukit Merah Reservoir, Perak, Malaysia. Trop Life Sci Res. 2018;29:103–17. 10.21315/tlsr2018.29.2.8 PMC6072723 30112144

[12] Kaliniak-Dziura A, Skałecki P, Florek M et al. Chemical composition and elements concentration of fillet, spine and bones of common carp (*Cyprinus carpio*) in relation to nutrient requirements for minerals. Animals. 2024;14:1311. 10.3390/ani14091311 38731315 PMC11083427

[13] Rasal KD, Kumar PV, Risha S et al. Genetic improvement and genomic resources of important cyprinid species: status and future perspectives for sustainable production. Front Genet. 2024;15:1398084. 10.3389/fgene.2024.1398084 39364006 PMC11446788

[14] Schuijt LM, Peng FJ, van den Berg SJP et al. (Eco)toxicological tests for assessing impacts of chemical stress to aquatic ecosystems: facts, challenges, and future. Sci Total Environ. 2021;795:148776. 10.1016/j.scitotenv.2021.148776 34328937

[15] Satam H, Joshi K, Mangrolia U et al. Next-generation sequencing technology: current trends and advancements. Biology (Basel). 2023;12:997. 37508427 10.3390/biology12070997 PMC10376292

[16] Ozsolak F, Milos PM. RNA sequencing: advances, challenges and opportunities. Nat Rev Genet. 2011;12:87–98. 10.1038/nrg2934 21191423 PMC3031867

[17] Wang Z, Gerstein M, Snyder M. RNA-seq: a revolutionary tool for transcriptomics. Nat Rev Genet. 2009;10:57–63. 10.1038/nrg2484 19015660 PMC2949280

[18] Li Y, Lv Y, Cheng P et al. Expression profiles of housekeeping genes and tissue-specific genes in different tissues of Chinese sturgeon (*Acipenser sinensis*). Animals. 2024;14:3357. 10.3390/ani14233357 39682323 PMC11639794

[19] Mohamed AR, King H, Evans B et al. Multi-tissue transcriptome profiling of north american derived atlantic salmon. Front Genet. 2018;9:369. 10.3389/fgene.2018.00369 30271423 PMC6146974

[20] Liao X, Cheng L, Xu P et al. Transcriptome analysis of Crucian carp (*Carassius auratus*), an important aquaculture and hypoxia-tolerant species. PLoS One. 2013;8:e62308. 10.1371/journal.pone.0062308 23630630 PMC3632525

[21] Salem M, Paneru B, Al-Tobasei R et al. Transcriptome Assembly, gene annotation and tissue gene expression atlas of the rainbow trout. PLoS One. 2015;10:e0121778. 10.1371/journal.pone.0121778 25793877 PMC4368115

[22] Chikina MD, Huttenhower C, Murphy CT et al. Global prediction of tissue-specific gene expression and context-dependent gene networks in *Caenorhabditis elegans*. PLoS Comput Biol. 2009;5:e1000417. 10.1371/journal.pcbi.1000417 19543383 PMC2692103

[23] Papatheodorou I, Fonseca NA, Keays M et al. Expression atlas: gene and protein expression across multiple studies and organisms. Nucleic Acids Res. 2018;46:D246–51. 10.1093/nar/gkx1158 29165655 PMC5753389

[24] Smith CM, Hayamizu TF, Finger JH et al. The mouse Gene Expression Database (GXD): 2019 update. Nucleic Acids Res. 2019;47:D774–9. 10.1093/nar/gky922 30335138 PMC6324054

[25] Raina P, Guinea R, Chatsirisupachai K et al. GeneFriends: gene co-expression databases and tools for humans and model organisms. Nucleic Acids Res. 2023;51:D145–58. 10.1093/nar/gkac1031 36454018 PMC9825523

[26] Guo C, Duan Y, Ye W et al. FishGET: a fish gene expression and transcriptome database with improved accuracy and visualization. iScience. 2023;26:106539. 10.1016/j.isci.2023.106539 37091248 PMC10119798

[27] Pasquier J, Cabau C, Nguyen T et al. Gene evolution and gene expression after whole genome duplication in fish: the PhyloFish database. BMC Genomics. 2016;17:368. 10.1186/s12864-016-2709-z 27189481 PMC4870732

[28] Sayers EW, Bolton EE, Brister JR et al. Database resources of the national center for biotechnology information. Nucleic Acids Res. 2022;50:D20–6. 10.1093/nar/gkab1112 34850941 PMC8728269

[29] Chang A, Jeske L, Ulbrich S et al. BRENDA, the ELIXIR core data resource in 2021: new developments and updates. Nucleic Acids Res. 2021;49:D498–508. 10.1093/nar/gkaa1025 33211880 PMC7779020

[30] Andrews S . FastQC: A quality control tool for high throughput sequence data.. Babraham Bioinformatics, Babraham Institute, Cambridge, UK., 2010.

[31] Bolger AM, Lohse M, Usadel B. Trimmomatic: a flexible trimmer for Illumina sequence data. Bioinformatics. 2014;30:2114–20. 10.1093/bioinformatics/btu170 24695404 PMC4103590

[32] Kim D, Paggi JM, Park C et al. Graph-based genome alignment and genotyping with HISAT2 and HISAT-genotype. Nat Biotechnol. 2019;37:907–15. 10.1038/s41587-019-0201-4 31375807 PMC7605509

[33] Li H, Handsaker B, Wysoker A et al. The sequence alignment/map format and SAMtools. Bioinformatics. 2009;25:2078–79. 10.1093/bioinformatics/btp352 19505943 PMC2723002

[34] Pertea M, Pertea GM, Antonescu CM et al. StringTie enables improved reconstruction of a transcriptome from RNA-seq reads. Nat Biotechnol. 2015;33:290–95. 10.1038/nbt.3122 25690850 PMC4643835

[35] Kang YJ, Yang DC, Kong L et al. CPC2: a fast and accurate coding potential calculator based on sequence intrinsic features. Nucleic Acids Res. 2017;45:W12–6. 10.1093/nar/gkx428 28521017 PMC5793834

[36] Condon K . tispec: Calculates Tissue Specificity from RNA-Seq Data. 2020.https://rdrr.io/github/roonysgalbi/tispec/ (10 January 2025, date last accessed).

[37] Moore B, Herrera M, Gairin E et al. The chromosome-scale genome assembly of the yellowtail clownfish *Amphiprion clarkii* provides insights into the melanic pigmentation of anemonefish. G3: Genes, Genomes, Genetics. 2023;13:jkad002. 10.1093/g3journal/jkad002 36626199 PMC9997566

[38] Leek JT, Johnson WE, Parker HS et al. The sva package for removing batch effects and other unwanted variation in high-throughput experiments. Bioinformatics. 2012;28:882–83. 10.1093/bioinformatics/bts034 22257669 PMC3307112

[39] Johnson WE, Li C, Rabinovic A. Adjusting batch effects in microarray expression data using empirical Bayes methods. Biostatistics. 2007;8:118–27. 10.1093/biostatistics/kxj037 16632515

[40] Kryuchkova-Mostacci N, Robinson-Rechavi M. A benchmark of gene expression tissue-specificity metrics. Brief Bioinform. 2017;18:205–14. 10.1093/bib/bbw008 26891983 PMC5444245

[41] Sánchez-Sevilla JF, Vallarino JG, Osorio S et al. Gene expression atlas of fruit ripening and transcriptome assembly from RNA-seq data in octoploid strawberry (Fragaria × ananassa). Sci Rep. 2017;7:13737. 10.1038/s41598-017-14239-6 29062051 PMC5653846

[42] Santos A, Tsafou K, Stolte C et al. Comprehensive comparison of large-scale tissue expression datasets. bioRxiv. 2014. 10.1101/010975 PMC4493645 26157623

[43] Durkin SM, Ballinger MA, Nachman MW. Tissue-specific and cis-regulatory changes underlie parallel, adaptive gene expression evolution in house mice. PLoS Genet. 2024;20:e1010892. 10.1371/journal.pgen.1010892 38306396 PMC10866503

[44] Petryszak R, Burdett T, Fiorelli B et al. Expression Atlas update—a database of gene and transcript expression from microarray- and sequencing-based functional genomics experiments. Nucleic Acids Res. 2014;42:D926–32. 10.1093/nar/gkt1270 24304889 PMC3964963

[45] Luigi-Sierra MG, Guan D, López-Béjar M et al. A protein-coding gene expression atlas from the brain of pregnant and non-pregnant goats. Front Genet. 2023;14:1114749. 10.3389/fgene.2023.1114749 37519888 PMC10382233

[46] Yao Y, Liu S, Xia C et al. Comparative transcriptome in large-scale human and cattle populations. Genome Biol. 2022;23:176. 10.1186/s13059-022-02745-4 35996157 PMC9394047

[47] Conesa A, Götz S, García-Gómez JM et al. Blast2GO: a universal tool for annotation, visualization and analysis in functional genomics research. Bioinformatics. 2005;21:3674–76. 10.1093/bioinformatics/bti610 16081474

[48] Kanehisa M, Furumichi M, Tanabe M et al. KEGG: new perspectives on genomes, pathways, diseases and drugs. Nucleic Acids Res. 2017;45:D353–61. 10.1093/nar/gkw1092 27899662 PMC5210567

[49] Emms DM, Kelly S. OrthoFinder: solving fundamental biases in whole genome comparisons dramatically improves orthogroup inference accuracy. Genome Biol. 2015;16:157. 10.1186/s13059-015-0721-2 26243257 PMC4531804

[50] Zhang H, Wang H, Shen X et al. The landscape of regulatory genes in brain-wide neuronal phenotypes of a vertebrate brain. eLife. 2021;10:e68224. 10.7554/eLife.68224 34895465 PMC8769648

[51] García-García L, Fernández-Tabanera E, Cervera ST et al. The transcription factor FEZF1, a direct target of EWSR1-FLI1 in Ewing sarcoma cells, regulates the expression of neural-specific genes. Cancers (Basel). 2021;13:5668. 34830820 10.3390/cancers13225668 PMC8616448

[52] Shimizu T, Nakazawa M, Kani S et al. Zinc finger genes *Fezf1* and *Fezf2* control neuronal differentiation by repressing *Hes5* expression in the forebrain. Development. 2010;137:1875–85. 10.1242/dev.047167 20431123

[53] Wang Z, Nakayama Y, Tsuda S et al. The role of gastrulation brain homeobox 2 (gbx2) in the development of the ventral telencephalon in zebrafish embryos. Differentiation. 2018;99:28–40. 10.1016/j.diff.2017.12.005 29289755

[54] Juárez-Morales JL, Weierud F, England SJ et al. Evolution of *lbx* spinal cord expression and function. Evol Dev. 2021;23:404–22. 10.1111/ede.12387 34411410 PMC8552994

[55] Tutukova S, Tarabykin V, Hernandez-Miranda LR. The role of neurod genes in brain development, function, and disease. Front Mol Neurosci. 2021;14:662774. 34177462 10.3389/fnmol.2021.662774 PMC8221396

[56] Voglsanger LM, Read J, Ch’ng SS et al. Differential level of RXFP3 expression in dopaminergic neurons within the arcuate nucleus, dorsomedial hypothalamus and ventral tegmental area of RXFP3-Cre/tdTomato mice. Front Neurosci. 2021;14:594818. 10.3389/fnins.2020.594818 33584175 PMC7873962

[57] Ma Y, Lv H, Wang J et al. Heterozygous mutation of *SLC34A1* in patients with hypophosphatemic kidney stones and osteoporosis: a case report. J Int Med Res. 2020;48:300060519896146. 10.1177/0300060519896146 32216560 PMC7133400

[58] Schubert FR, Dietrich S, Mootoosamy RC et al. Lbx1 marks a subset of interneurons in chick hindbrain and spinal cord. Mech Dev. 2001;101:181–5. 10.1016/S0925-4773(00)00537-2 11231071

[59] Alayoubi AM, Alfadhli F, Mehnaz et al. A homozygous variant in ARHGAP39 is associated with lethal cerebellar vermis hypoplasia in a consanguineous Saudi family. Sci Rep. 2024;14:25291. 10.1038/s41598-024-77541-0 39455833 PMC11511811

[60] Jin L, Li Y, Luo S et al. Recessive APC2 missense variants associated with epilepsies without neurodevelopmental disorders. Seizure. 2023;111:172–77. 10.1016/j.seizure.2023.08.008 37657306

[61] Montalbano G, Levanti M, Mhalhel K et al. Acid-sensing ion channels in zebrafish. Animals. 2021;11:2471. 10.3390/ani11082471 34438928 PMC8388743

[62] Männik K, Arbogast T, Lepamets M et al. Leveraging biobank-scale rare and common variant analyses to identify *ASPHD1* as the main driver of reproductive traits in the 16p11.2 locus. bioRxiv. 2019. 10.1101/716415

[63] Cai Y, Lv W, Jiang Y et al. Molecular evolution of the BRINP and ASTN genes and expression profiles in response to pathogens and spinal cord injury repair in lamprey (Lethenteron reissneri). Fish Shellfish Immunol. 2022;131:274–82. 10.1016/j.fsi.2022.09.076 36228880

[64] Soni S, Makwana SH, Bansal S et al. Lipid metabolism associated PLPP4 gene drives oncogenic and adipogenic potential in breast cancer cells. Biochim Biophys Acta Mol Cell Biol Lipids. 2025;1870:159609. 10.1016/j.bbalip.2025.159609 40187483

[65] Kumar A, Torii T, Ishino Y et al. The Lewis X-related α1,3-fucosyltransferase, Fut10, is required for the maintenance of stem cell populations. J Biol Chem. 2013;288:28859–68. 10.1074/jbc.M113.469403 23986452 PMC3789981

[66] Dragojević J, Mihaljević I, Popović M et al. In vitro characterization of zebrafish (*Danio rerio*) organic anion transporters Oat2a-e. Toxicol In Vitro. 2018;46:246–56. 10.1016/j.tiv.2017.09.026 29030288

