# Supplementary material for: CyExpDB: a web-based multi-species tissue-specific gene expression platform for functional genomics in Cyprinidae fish
Source: Database (Oxford). 2025 Dec 31;2025:baaf087. doi: 10.1093/database/baaf087 (PMC12754418; doi:10.1093/database/baaf087)

**Supplementary Figure S1.** Principal Component Analysis (PCA) of normalized gene expression profiles for five Cyprinidae species: (A) *Cyprinus carpio*, (B) *Labeo rohita*, (C) *Carassius gibelio*, (D) *Carassius auratus*, and (E) *Ctenopharyngodon idella*. For each species, PCA plots colored by tissue (left) and BioProject (right) show clear tissue-specific clustering and absence of batch effects, confirming effective correction with ComBat.

**(A)** *Cyprinus carpio*

*
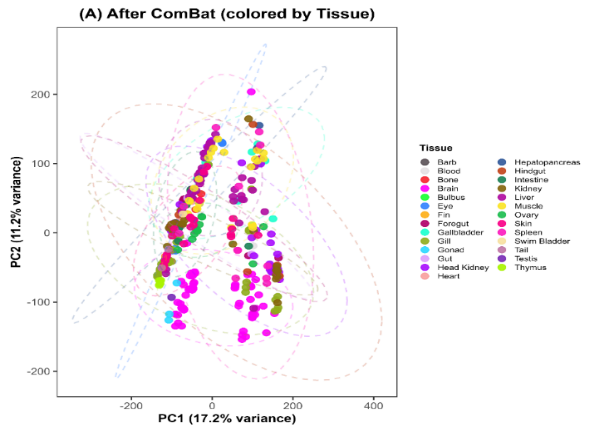
*
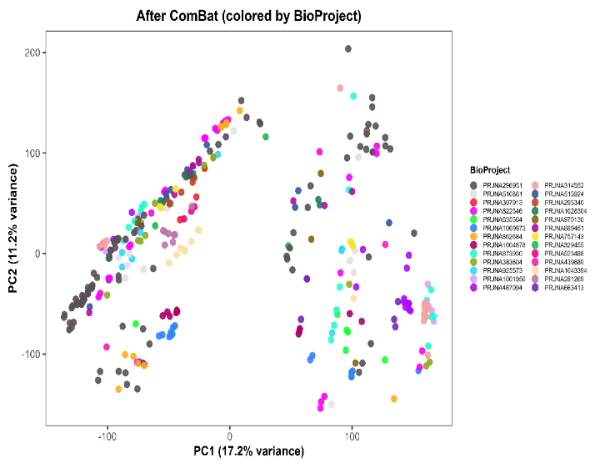


**(B)** *Labeo rohita*

*
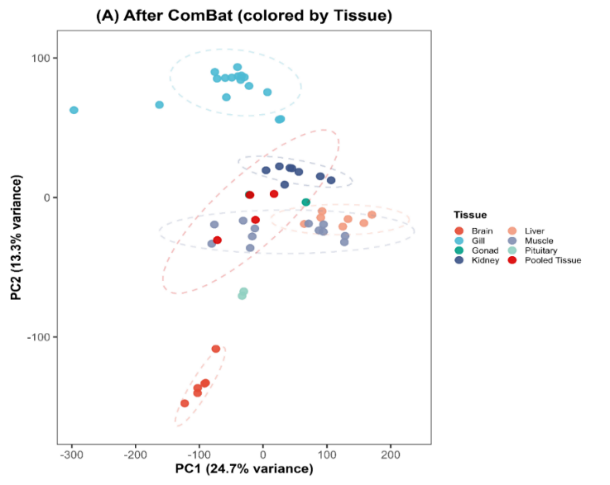

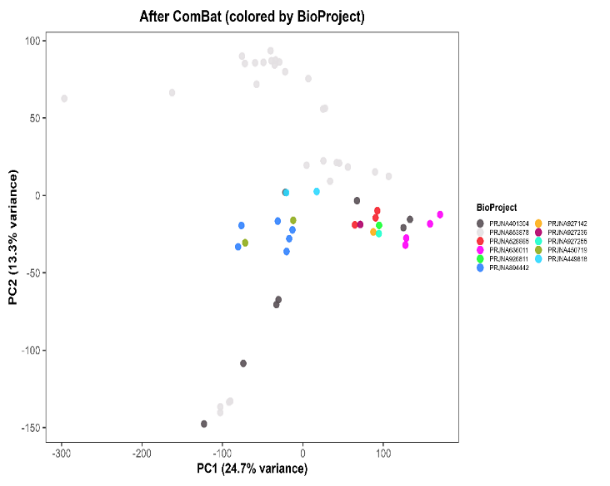
*


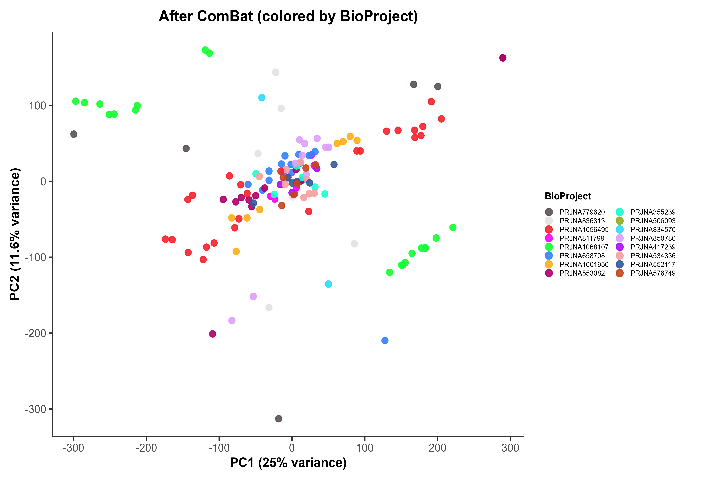
**(C)** *Carassius gibelio*


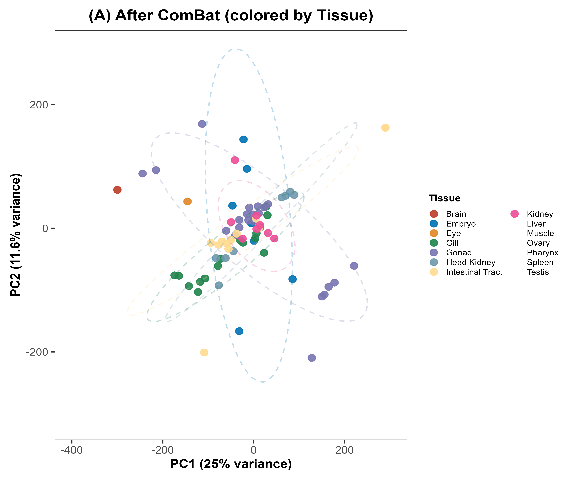


***
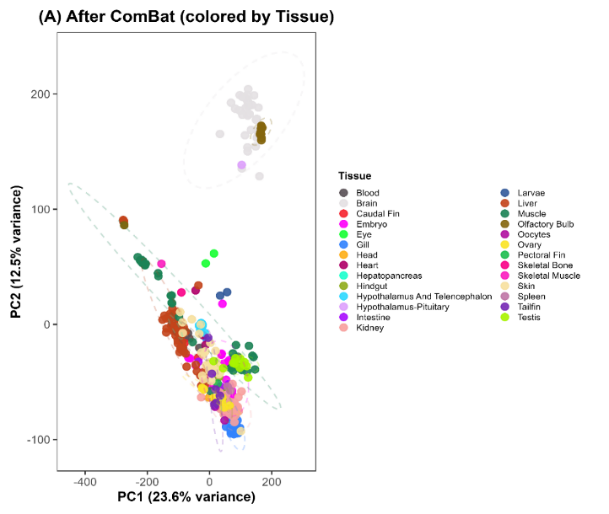
*(D)** *Carassius auratus*

**
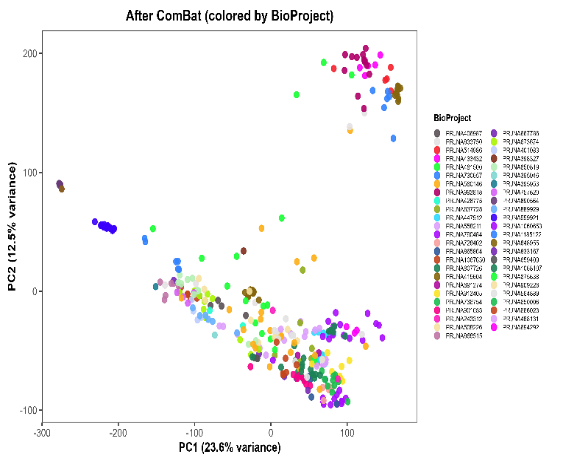
**

**(E)** *Ctenopharyngodon idella*


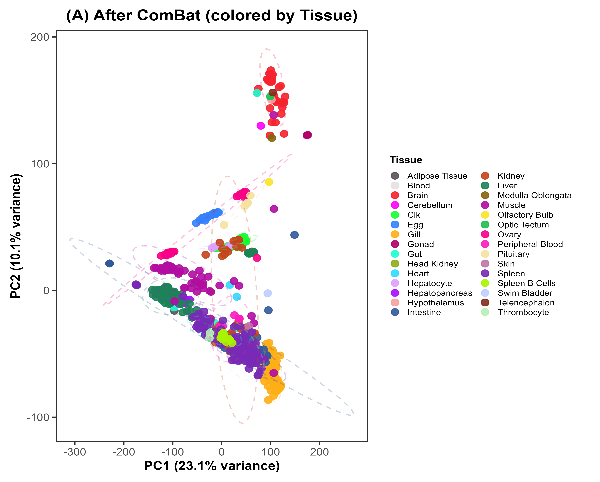
**
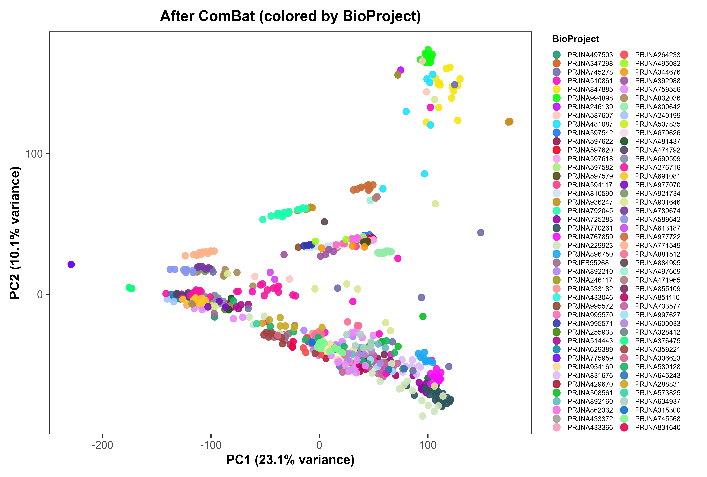
**

**Supplementary Figure S2.** Sensitivity analysis of the tissue-specificity index (τ) to normalization strategies across Cyprinidae species.
Representative scatter plots illustrate the concordance of τ values derived from different normalization methods, FPKM and TPM, for (A) *Cyprinus carpio*, (B) *Labeo rohita*, (C) *Carassius gibelio*, (D) *Carassius auratus*, and (E) *Ctenopharyngodon idella*.

**(A)** *Cyprinus carpio*

**
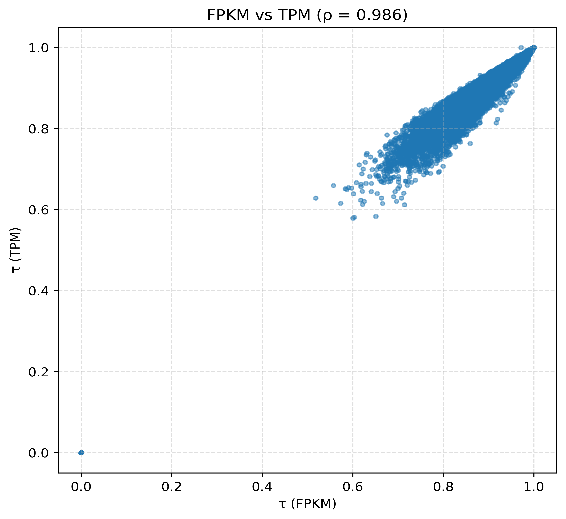
**

**(B)** *Labeo rohita*


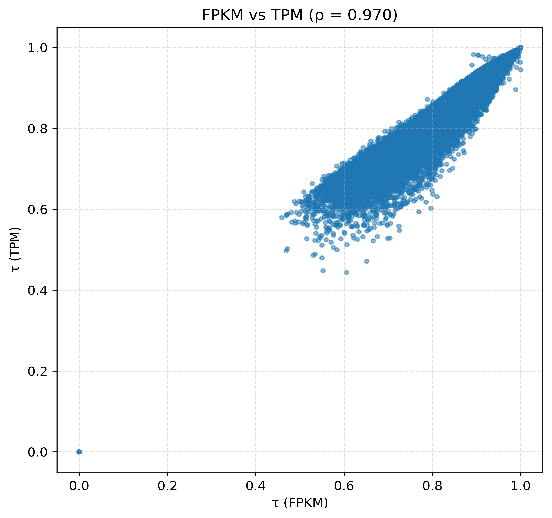


**(C)** *Carassius gibelio*


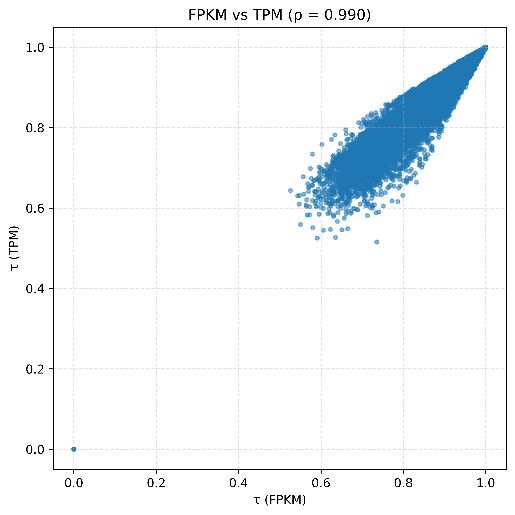


**(D)** *Carassius auratus*


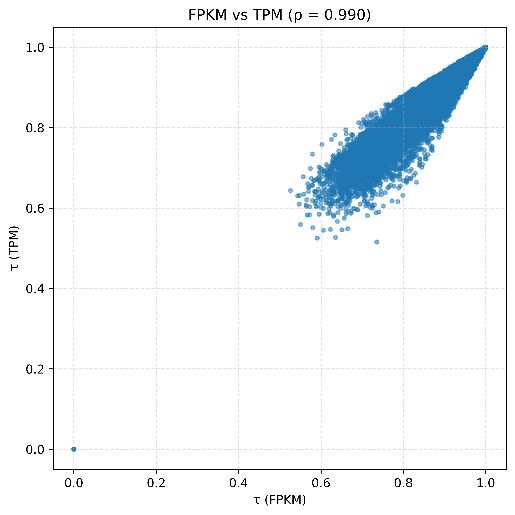


**(E)** *Ctenopharyngodon idella*


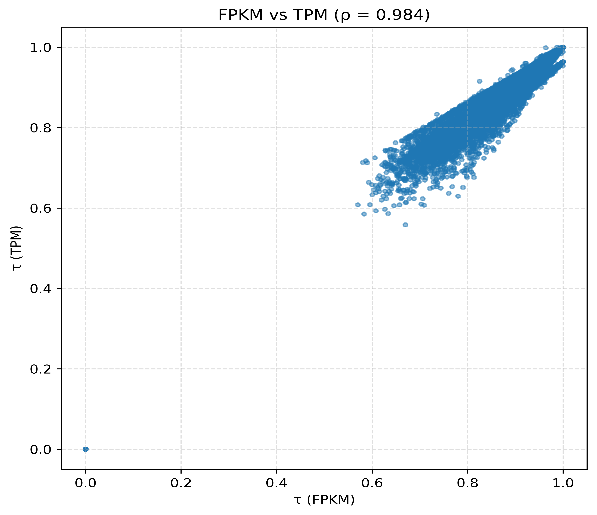

Supplement: baaf087_Supplemental_Files [file baaf087_supplemental_files.zip › Supplementary Figure.docx]
